# Curious Case of Monomania

**Published:** 1860-11

**Authors:** 


*Curious Case of Monomania.*—The following, if not a *canard*, is a curious case of monomania:

A citizen of Berlin, a man in comfortable circumstances, is periodically attacked with a desire to knock off hats. He afterwards makes up the loss to the astonished victim of this strange fancy by the payment of three thalers. According to the calculation of his family, in the past year he has been obliged to make good the loss of two hundred and sixty-seven hats. At a recent musical festival, fifty-three hats were sacrificed to this curious frenzy, and for the evening's entertainment he paid a hundred and fifty-nine thalers. —*Med. & Surg. Reporter.*

---

*Army and Navy.*—Surgeon R. H. Coolidge will be relieved from duty in the Surgeon-General's Office on the first of December next, and will then proceed, via Panama, to the Head-Quarters of the Department of California, and report for duty, as Medical Surveyor for the Departments of California and Oregon. Surgeon Coolidge will be assigned to Benicia Barracks.

Surgeon R. Murray will be relieved from duty in the Department of California on the arrival of Surgeon Coolidge, with directions to repair to Baltimore, Md., and report thence by letter, to the Surgeon-General for further orders.

Assistant Surgeon C. H. Smith has been assigned to duty in the Surgeon-General's Office, and ordered to report in person to the Surgeon-General as soon as his present duties in Baltimore are completed.—*Reporter.*

☞ Clinical instruction in Bellevue Hospital, New York, has, by order of the Commissioners, been made free to students, almost at the same time that a similar course was adopted by the Board of the Philadelphia Hospital. This makes all the Hospitals of New York accessible to students free of charge. It is, we think, high time that the Pennsylvania Hospital should follow.

